# Ecological Beeswax Breast Pads Promote Breastfeeding in First-Time Mothers from the Valencian Community (Spain): A Randomized Trial

**DOI:** 10.3390/healthcare13111330

**Published:** 2025-06-03

**Authors:** Irene Pastor-Pagés, Verónica Ausina-Marquez, María Mercedes Rizo-Baeza, Ernesto Cortés-Castell, Ana Noreña-Peña

**Affiliations:** 1 Faculty of Health Sciences, European University of Valencia, 46010 Valencia, Spain; irenepastorpages@gmail.com; 2 Nursing Department, Universidad de Alicante, 03690 San Vicent del Raspeig, Spain; rizo.mercedes@gmail.com (M.M.R.-B.); ana.norena@ua.es (A.N.-P.); 3 Pharmacology, Pediatric and Organic Chemistry Department, University Miguel Hernández de Elche, 03550 San Juan de Alicante, Spain; ernesto.cortes@umh.es

**Keywords:** breastfeeding, breast pads, health education

## Abstract

Introduction. Scientific societies emphasize the benefits of breastfeeding. The effectiveness of education, information, and support during pregnancy has been demonstrated. However, further research is needed on the prevention and treatment of nipple damage, which is a major cause of breastfeeding cessation. Objective. To determine whether the use in clinical practice of beeswax breast pads in combination with the WHO (World Health Organization) health education program improves continuation of breastfeeding among first-time mothers over a 6-month follow-up period compared with the use of the WHO health education program alone. Material and methods. A prospective randomized control study was conducted in 122 first-time mothers who were seen in the midwifery office between 2017 and 2021 in several health centers in Alicante and Valencia. Data were collected through a prepartum health questionnaire, in the first weeks postpartum, and at 3 and 6 months postpartum. Results. A higher percentage of mothers in the intervention group continued breastfeeding at 6 months compared to the control group (76.3% versus 57.1%). The continuity of breastfeeding is only significant in relation to the use of the breast areolas, with values, respectively, at 3 and 6 months OR (odds ratio) = 3.129 (95% CI; 1.249–7.839; *p* = 0.015) and OR = 2.282 (95% CI, 1.038–5.016; *p* = 0.040). The NNT (number needed to treat) at 3 months = 5 (95% CI 3–13; *p* = 0.004) and at 6 months = 5 (95% CI 3–26; *p* = 0.025) Conclusions. The use of organic beeswax breast pads in combination with the WHO health education program improves initiation and maintenance of exclusive breastfeeding among first-time mothers and the perception of their health status compared to the health education program alone.

## 1. Introduction

The World Health Organization (WHO [1]) and other scientific societies recommend exclusive breastfeeding (EBF) until 6 months of age followed by breastfeeding together with complementary foods until the mother or child chooses.

In the meta-analysis by Thomson et al. [2], a significant association between breastfeeding and protection against sudden infant death was found only in infants who were breastfed for more than two months, with greater protection associated with longer duration of breastfeeding. Similarly, in a meta-analysis of gut microbiota by Ho et al. [3], a more stable gut microbiota was observed in infants receiving EBF for at least 2 months.

Mothers need support for both the initiation and maintenance of breastfeeding, which is why strategies to increase breastfeeding rates include promotion and education. Some reviews conclude that breastfeeding promotion can increase breastfeeding in the short and long term, and is most effective before and immediately after childbirth, rather than waiting for problems to arise in initiating and maintaining breastfeeding [4,5].

Combined individual and group counseling has also been shown to be more effective than either one alone [6]. Recently, a positive association has been found between the promotion of breastfeeding in the hospital and rooming-in with its duration [7,8], as well as previous training of mothers [9]. The clinical trial by Dagla et al. [10] examined a sample of 1080 Greek women who had received a health intervention. This study showed that three important research assumptions were in practice, which are internationally associated with increased initiation and continuation rates of breastfeeding: (a) midwifery-led antenatal education/counselling for the pregnant woman and her partner; (b) midwifery-led continuous long-term support, counselling, and monitoring of breastfeeding during the puerperium and lactation period; and (c) early detection of symptoms of mental health disorders and early psychosocial intervention. Of the women, 96.3% initiated breastfeeding, and by the end of the sixth month postpartum, 44.3% of the infants were exclusively breastfed. Greater counseling by midwives was correlated with a higher likelihood of EBF at 6 months postpartum (*p* = 0.034) and with a longer duration of breastfeeding (*p* = 0.015).

The Cochrane review by Britton et al. [11] included 34 studies with a sample of 29,385 breastfeeding women, evaluating all forms of breastfeeding support. All interventions by both health and non-health providers increased breastfeeding rates (including mixed breastfeeding and EBF). EBF was significantly increased by the WHO/UNICEF [12] training program (RR 0.69, 95% CI 0.52 to 0.91).

Among the causes of breastfeeding cessation, Aldalili and El Mahalli [13] found discomfort/fatigue from breastfeeding as an associated risk factor at 1 month, 2 months, and 6 months, and nipple soreness as an associated risk factor at 2 months and 6 months. Nipple cracking, mastitis, and breast engorgement are the most common breast problems when breastfeeding [14]. Their incidence is between 34 and 96%, and when they occur, one-third of mothers stop breastfeeding [15,16,17].

An adequate latch, with a good seal between the infant’s mouth and the mother’s breast, is the basis for preventing breast complications such as cracked nipples, engorgement, and mastitis. Among the treatments used to heal cracked nipples, greater effectiveness was found with the use of lanolin, dexpanthenol-based creams, breast protectors, and the application of mint. Direct application of breast milk was also shown to be the most economical intervention, with very good results, except for an increase in healing time [18,19].

According to the latest data published by the Spanish National Statistics Institute (INE) [20], the prevalence rate of EBF in the Valencian Community is 25.61% at 6 months, with total breastfeeding (including mixed breastfeeding) at 40.14%. Slightly higher rates were found at the national level, placing the EBF rate at 35.2% [21]. The WHO breastfeeding targets for 2025 are to ensure that more than half of all newborns are breastfed until at least 6 months of age in order to reduce infant mortality and sudden infant death syndrome rates [22].

Based on the review of the literature and the demonstrated effectiveness of education to increase the duration of EBF, it is necessary to conduct more studies on the treatment and protection of nipple injuries, as this is one of the main causes of discontinuation of breastfeeding. Beeswax breast pads can soothe sensitive and dry nipples by avoiding friction with the tissues and keeping them hydrated by retaining drops of milk and traces of baby saliva, preventing the formation of cracks and other discomfort typical of breastfeeding, due to its antiseptic, healing, and anti-inflammatory properties [23].

It has also been shown that virgin beeswax is a natural product that has properties for skin care, creating a semi-occlusive skin barrier that minimizes transepidermal water loss, acting as a moisturizer, and relieving symptoms associated with skin problems such as dermatitis, psoriasis, and the development of normal skin flora [24].

For this reason, we set the following objectives: To determine whether the use in clinical practice of virgin beeswax breast pads combined with the WHO health education program improves anxiety and depression levels and self-perception of health status and the initiation and continuation of EBF among first-time mothers over a 6-month follow-up period, compared with the use of the WHO health education program alone.

We hypothesize that the use of organic virgin beeswax breast pads improves the sensation of pain and nipple problems in the mother, and, in combination with the WHO health education program, improves initiation and maintenance of EBF in first-time mothers compared to the health education program alone.

## 2. Methods

To achieve the objectives, we designed a prospective analytical experimental study, in which a comparative randomized intervention study was conducted between two groups of first-time mothers. This study is registered in the Clinical Trials with the number NCT03676608 and title: Study of Health Education to Improve Adherence to Breastfeeding in Primiparous Women Through the Use of Bee Wax Mammary Areolae.

The study group consisted of 122 first-time mothers with the intention of breastfeeding their children, residing in the Valencian Community. The study was carried out between 2017 and 2022. The volunteers were randomly assigned to the control or intervention group during the antepartum visits to the midwife, using a random number generator and following the order of arrival at the office of the patients until the required number was completed. The groups were the control group, women who received the WHO education program to promote breastfeeding, and the intervention group, which followed a mixed strategy using the WHO program plus the use of beeswax breast pads.

### 2.1. Inclusion Criteria

- First-time mothers during the third trimester of pregnancy with a desire to breastfeed and who signed the informed consent.
- Only participants who attended the WHO educational program and those in the intervention group used the breast areolas daily.

### 2.2. Exclusion Criteria

- Before starting the study, exclusion criteria were established: atopy and/or dermatological problems; mental health conditions; allergies to bee products; and not speaking or understanding Spanish.
- The following cases were eliminated as exclusion criteria, although they were not designed as exclusion criteria: pregnant woman who did not go to the scheduled hospital to give birth; hospitalization of the newborn for any reason; and did not attend the midwife’s office during the days following postpartum discharge and were considered losses.

### 2.3. Sampling Size

With an approximate percentage of breastfeeding at six months of 40% according to the previous data and an increase up to 50% marked as success, with a risk level of 0.05 and a statistical power of 90%, and considering that we will only accept an increase due to the use of the areolas (and therefore it is a unilateral test), 40 cases per group were calculated. Considering that about 20% of mothers may be lost during the follow-up of the study, the sample for each group should be 48 [25].

### 2.4. Intervention

The pregnancy care program was taught to both groups equally, starting before random assignment to the control or intervention group. It was carried out during 6 group theoretical/practical sessions, lasting two hours, taught by the primary care midwife from 30 weeks of gestation, following international recommendations on the functions of the midwife [26]. During these sessions and in relation to breastfeeding, aspects such as presentation of the recommendations of the WHO [12,22] and UNICEF [27] were addressed in relation to the start before the first hour of life and duration of at least 6 months of breastfeeding, BF, milk composition, breast function and physiology of milk production, most frequent problems during BF, signs of a good latch as well as postural recommendations to achieve it, difficulties in the technique and causes of a poor latch, reasons that make it necessary to express milk and how to perform it, time and way to preserve breast milk for later use, and recommendations for returning to work. With the arrival of COVID-19 and the suspension of all group activities in health centers, an audiovisual document was prepared with the same information and sent to each study participant. Furthermore, all this information was reinforced with individual prepartum, postpartum, 3-month, and 6-month consultations by the midwife.

Assessments were performed at 36 weeks gestation; they were given the prepartum questionnaire to collect information on the type of sample we had and the secondary variables, as well as in the first few weeks postpartum, at 3 months postpartum, and 6 months postpartum. The women in the intervention group began to apply the beeswax breast pads at least 1 h per day from week 38, and we asked the patients in the various interviews about its use. A pair of the beeswax breast pads can be used throughout the breastfeeding period, with a priority use date of 2 years.

### 2.5. Main Variable

Continuation of EBF at 3 and 6 months (the infant receives only breast milk as food).

*Secondary variables:* maternal age, type of delivery, breast complications, gestational weeks at delivery, time between delivery and initiation of breastfeeding, and need for latch-on assistance. In addition, we used the Euroqol-5D quality of life test, used by different authors in pregnant and postpartum women [28], structured in five dimensions: mobility, personal care, daily activities, pain/discomfort, and anxiety/depression, with responses of none (1), some (2) and a lot (3); the highest score is the worst. The visual self-perception scale is measured from 0 to 100, where the higher score indicates better self-perception of one’s state of health. In addition, we used the Golberg test with its two dimensions, anxiety and depression, structured in 18 questions with a yes (1) and no (0) response; the state of anxiety/depression is indicated as worse with a higher score.

Funding sources: The company provided the beeswax breast pads free of charge, which was the only source of funding.

### 2.6. Description of the Beeswax Discs (Mamaceram®)

**Figure 1:**
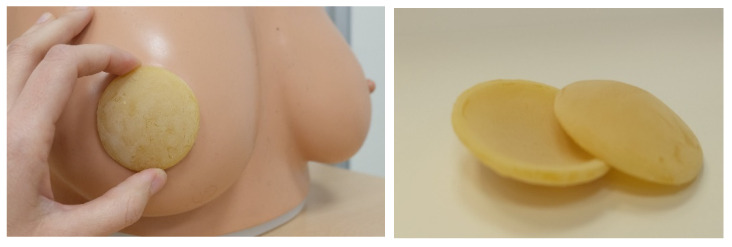
Image and placement of the ecological beeswax breast pads.

The organic beeswax discs used as breast areolas are made by pouring approximately 9 g of pure beeswax, melted in a bath at 65 °C, into suitable molds and allowing it to cool until it acquires the natural solid consistency of wax. The result is thin, rigid discs that cover the areola and nipple of the breast directly against the skin (Figure 1).

## 3. Ethical Issues

This study was approved by the Clinical Research Ethics Committee of the Sagunto Health Department (Valencia) on 26 March 2020 with reference number 202008-PIB-TED and by the Elche Health Department (Alicante) on 30 November 2016; it is in compliance with the fundamental rights established in the Declaration of Helsinki and the Data Protection Act.

## 4. Statistical Analysis

A descriptive analysis was performed with the qualitative variables expressed as absolute frequency (n) and relative frequency (%) and the quantitative variables expressed as mean and standard deviation. Qualitative variables were compared between the two groups analyzed using the chi-square test, and quantitative variables were compared using the Student’s *t*-test. To eliminate possible confounding variables, a binary logistic regression analysis was performed in relation to the maintenance of breastfeeding at 3 months and 6 months, compared to all the variables analyzed pre- and postpartum, with those that did not enter the regression model being eliminated through steps. From the mothers who continued breastfeeding in the control group and intervention group, the corresponding number needed to treat (NNT) with their 95% confidence intervals (95% CI) were calculated from the inverse of the absolute risk reduction (1/RAR) [25]. The level of significance was considered to be *p* < 0.05. All statistical analyses were performed using IBM SPSS statistics software, version 27 (New York, NY, USA).

## 5. Results

### Characteristics of the Participants

**Figure 2:**
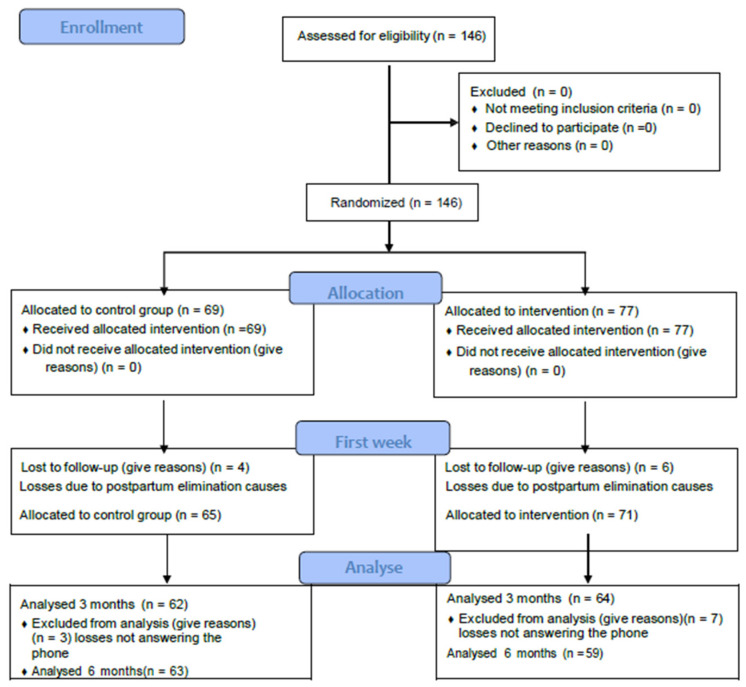
Consort flow diagram of the evolution of the analyzed cases.

A total of 146 women participated in the study, with a final sample comprising 122 first-time mothers (83.6%). Once the estimated necessary sample was exceeded, we finalized the collection of the sample. A flow chart of the evolution of the number of women included in the study is shown in Figure 2.

Analysis of the variables examined between the control and intervention groups showed no differences between the two groups at the time of delivery (Table 1), indicating that the two groups were well established by randomization.

The results and their comparison at postpartum are shown in Table 2, with no differences found between the two groups in any of the variables analyzed. Thus, the two groups remained homogeneous and, from this point on, the EBF follow-up between the two groups can be reliably compared.

In Table 2, it is worth highlighting a small postpartum increase, common in both groups, in depression on the Goldberg scale and in the dimensions of the EQ5D test and, on the contrary, a decrease in anxiety. There is also a slight decrease in self-perception and an increase in postpartum discomfort compared to prepartum, although none of the differences are significant.

When analyzing the values of the tests carried out after 3 months, an improvement is observed in all the indicators, with the differences in Goldberg’s anxiety and depression not being significant, but the improvement in self-perception is significant, being 81.8 ± 12.9 for the control group and 86.3 ± 12.2 for the intervention (*p* = 0.049); with the EQ5D mobility domain 1.1 ± 0.2 versus 1.0 ± 0 (*p* = 0.045) and the pain/discomfort domain with values 1.2 ± 0.4 versus 1.1 ± 0.2 (*p* = 0.046).

**Figure 3:**
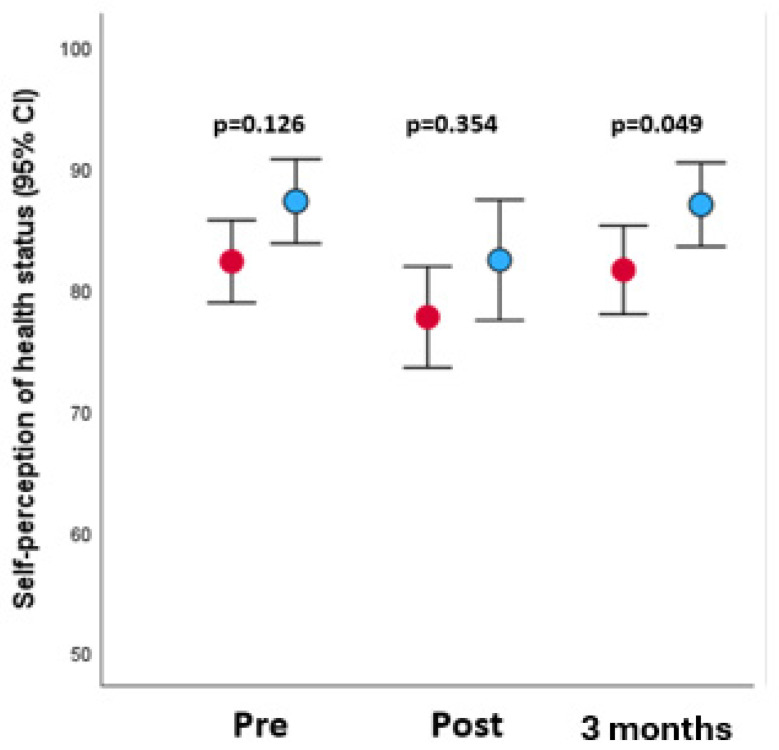
Evolution of the self-perception of the health status of the women studied (control group: red; intervention group: blue).

As an illustration of the mother’s self-perceived health status, its evolution during the study is presented (Figure 3), with no different values observed between the control and intervention groups, with a drop after delivery and a subsequent rise to previous levels in which a difference in better self-perception is observed among mothers who have used wax breast areolas.

This follow-up shows a higher percentage of mothers who continued breastfeeding at 3 and 6 months (Table 3).

From these distributions of mothers who continued breastfeeding in the control and intervention groups, the following results were obtained: at 3 months NNT = 5 (95% CI 3–13; *p* = 0.004) and at 6 months NNT = 5 (95% CI 3–26; *p* = 0.025), which means that placing the mammary areolas on five mothers prevents a loss of lactation that would occur in the control group.

**Figure 4:**
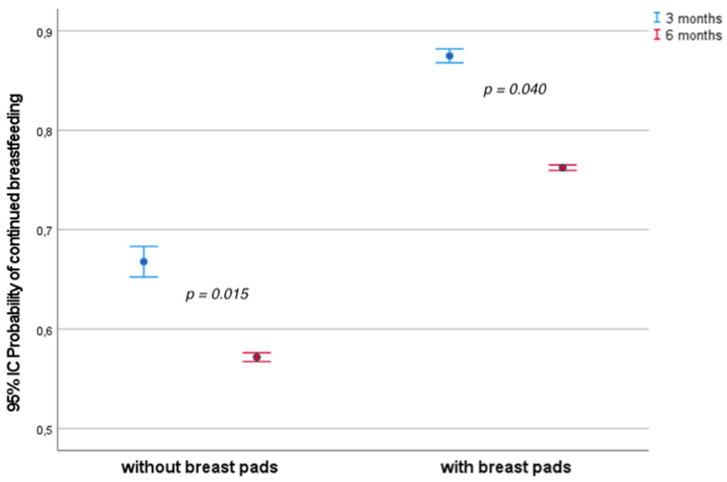
Predicted probability according to the binary logistic regression model of continued breastfeeding at 3 (blue, *p* = 0.015) and 6 months (red, *p* = 0.040) postpartum in the control and intervention groups.

A binary logistic regression analysis was performed with stepwise exclusion of variables until reaching the most significant model of mothers who continued breastfeeding at 3 and 6 months (Table 4). In both cases, the only variables that entered the model were the type of delivery and the use of beeswax breast pads, with use of breast pads being the significant variable in both periods (OR = 3.129 (95% CI; 1.249–7.839; *p* = 0.015) at 3 months and OR = 2.282 (95% CI, 1.038–5.016; *p* = 0.040) at 6 months). The regression models were used to obtain the probabilities of continued breastfeeding with and without the use of breast pads in both periods (3 and 6 months postpartum). These probabilities are presented in Figure 4, which shows an increase in the probability of continued breastfeeding in both periods when the breast pads and the education program were used together compared to the education program alone.

## 6. Discussion

Many studies have linked breastfeeding training and education to improved breastfeeding rates [10,23,27].

There are no updated official data on breastfeeding rates in Spain [29]. According to data from UNICEF [27], the global rate of EBF in infants under 6 months of age is 43%. Data published by Pérez-Escamilla et al. [30] report a rate of 48.6% in 2019. This is lower than the data obtained in our study among first-time mothers who breastfed at 3 months in both the intervention and control groups, with a rate of 87.7% and 66.7%, respectively, and a statistical significance of *p* = 0.004. This significant association was also observed at 6 months, with a 19.2% higher breastfeeding rate in the intervention group than in the control group, *p* = 0.025 (76.3% breastfeeding in the intervention group versus 57.1% in the control group).

Binary logistic regression analysis was performed on the variable of continued breastfeeding at 3 months. The two variables included in the model were type of delivery (vaginal), which was not significant (OR = 0.561; 95% CI, 0.211–1.490; *p* = 0.246), and the variable with the largest effect, use of breast pads (OR = 3.129; 95% CI, 1.249–7.839; *p* = 0.015), which was significant.

Based on this model, we calculated the probability of continued breastfeeding with and without the use of breast pads and found that the probability of continued breastfeeding at 3 months was almost 90% in the intervention group compared to almost 70% in the control group.

Similarly, at 6 months, the variables in the binary logistic regression model continued to be type of delivery (vaginal) (OR = 0.814; 95% CI, 0.324–2.045; *p* = 0.662) and the variable with the largest effect and significance, use of breast pads (OR = 2.282; 95% CI, 1.038–5.016; *p* = 0.040). After applying the logistic regression model, we calculated the probability of continued breastfeeding at 6 months and obtained a probability of less than 60% in the control group and greater than 75% in the intervention group.

In the clinical trial by Dagla et al. [10], applying prenatal and postnatal advice and during the first year postpartum and monitoring and support of breastfeeding and mental health by the midwife, as indicated in the introduction, demonstrated that this intervention was key to continued breastfeeding, achieving a breastfeeding rate of 44.3% at the end of 6 months postpartum. Counseling was correlated with a higher likelihood of EBF at 6 months postpartum (*p* = 0.034) and with a longer duration of breastfeeding (*p* = 0.015). The Best Practice Guideline—Breastfeeding Promoting and Supporting the Initiation, Exclusivity and Continuation of Breastfeeding for Newborns, Infants and Young Children also indicates that breastfeeding counseling should be included prenatally to encourage the breastfeeding process [26]. In addition, the systematic review by De la Hoz et al. [29] emphasizes the need for good prenatal and postnatal education, with an emphasis on correct latching to prevent nipple cracking. Accordingly, in our study, the WHO education program was implemented in all participants prenatally and then in the postpartum period. Breastfeeding was evaluated, latch-on was assessed, and the possible occurrence of complications was monitored in both groups. Haroon et al. [6] had already shown that this counseling was more effective when it was carried out both individually and in groups (including both). However, due to the onset of the COVID-19 pandemic, it was not possible to provide joint individual and group counseling to the entire sample collected in our study, as all group activities were suspended during the months of the pandemic.

A Cochrane review of 34 studies with a sample of 29,385 women concluded that all breastfeeding support interventions, whether delivered by health professionals or not, increased breastfeeding rates. The WHO/UNICEF training program led to an increase in months of breastfeeding (RR = 0.69; 95% CI 0.52–0.91) [11,12]. When we compare our results in the control group with the literature consulted, we find that we had better results, achieving a breastfeeding rate of over 50% (57.1%).

Renfrew et al. [15] found that breast complications (such as nipple cracking, breast engorgement, and mastitis) occur in 34–96% of women and that when they occur, one-third of mothers discontinue breastfeeding. These data are consistent with those found in our study, in which 68.9% of our sample experienced discomfort in the first few weeks postpartum, and of these, 26.65% stopped breastfeeding before the ninth week postpartum. This could be related to the anti-inflammatory and regenerative properties of beeswax [23]. However, this rate of discontinuation is lower than that reported by Renfrew et al. [15]. No significant differences were noted for the prevention of pain and nipple disorders with the use of lanolin (*p* = 0.61 and *p* = 0.21, respectively) [31]. However, the sample size was small with 66 participants. In the clinical practice guideline, they also did not obtain significance for the perception of breast pain in the mother with the application of glycerin patches, lanolin, and other ointments [32]. In the single-blind randomized controlled trial by Perić et al. [33] involving 206 first-time mothers with sore and damaged nipples, they concluded that both lanolin and breast milk itself were equally effective in treating these breast complications. They randomly assigned 103 women to the control group (breast milk application) and 103 women to the intervention group (lanolin application). They found, as in the study by De la Hoz et al. [29], that the healing time was longer with the application of breast milk. Nevertheless, they found no statistically significant differences for other secondary outcomes such as breastfeeding time [33]. In contrast, our results show a significant increase in the duration of breastfeeding in the intervention group, with a higher breastfeeding rate at 6 months in the breast pad group compared to the control group (76.3% versus 57.1% in the control group, *p* = 0.02). Likewise, Aguilar et al. [34], in their experimental study of 300 Cuban lactating mothers, 150 of whom used EVOO and 150 of whom applied drops of breast milk, found a significant association between the application of three drops of EVOO after each feeding and the absence of nipple cracking. As a result, they achieved a reduction in pain and a higher breastfeeding rate. They evaluated the presence of cracking during the first week of breastfeeding, in the second week, and after one month. A significant relationship (*p* < 0.05) was found in the first month, with a frequency of cracking of 2.7% in the EVOO group and 44% in the breast milk group. The same comparison was made by Agea-Cano et al. [35] with 124 women, at the San Juan de la Cruz Hospital in Ubeda, who were randomly divided into an intervention group (application of organic EVOO) and a control group (application of breast milk). The application in both groups was at least four times after feeding. No significant difference was found for the variable nipple soreness, but there was a significant difference for the appearance of cracking, which was significantly lower in the first-time mothers in the EVOO application group. However, this statistical relationship was not found in the multiparous women.

Similarly, in Egypt, they compared the use of EVOO with routine pharmacological treatment for nipple trauma as prescribed by a physician, and they obtained, with a statistically significant relationship, that mothers who used EVOO recovered faster from nipple trauma [36]. In the study of Abdelmawgoud Ahmed et al. [37], the extra virgin olive oil group had significantly less teat pain and teat trauma, as well as better healing of teat trauma compared to the breast milk group at days 3, 7 and 14 (*p* value < 0.01).

In the same line, Perić et al. [33] conducted an experimental study comparing three groups (1: EVOO, 2: breast milk, and 3: control) and assessed them on days 3, 7, and 14. The EVOO group was asked to clean and dry their breasts after breastfeeding and then apply the oil so as not to introduce a confounding factor with the milk that might remain on the nipple and areola. They found that nipple pain on day 14 was significantly lower in groups 1 and 2 than in the control group (group 3) (*p* = 0.001), and that only the EVOO group had a significant decrease in the mean pain score on all days of follow-up. They found no significant differences between groups 1 and 2 (EVOO and breast milk) and the appearance of cracks, but they note the sample size as a limitation.

## 7. Limitations

We selected only first-time mothers who intended to breastfeed, excluding from the study those who clearly stated their intention not to breastfeed, although these were a minority and only women residing in the Valencian Community (Spain).

Another important limitation was the pandemic situation caused by COVID-19, which led to a change in health care delivery. All group activities were suspended, including prenatal maternal education workshops and postnatal breastfeeding workshops. This meant that we had to redirect our study and produce an audiovisual document to implement the WHO breastfeeding training program. As a result, part of our sample received the program in person in group sessions and another part received it remotely on an individual basis, although analysis of the behavior under both conditions revealed no differences.

This change in health care delivery also affected the sample size, both for the initial sample and for those who dropped out due to loss of contact, although the rate of loss was not different from that of other follow-up studies: of 146 who initially participated, 24 (16.4%) were lost. The main strength of the study is that, at a very low cost, it is possible to achieve a notable increase in breastfeeding duration up to 3 and 6 months. In addition, the results are very reliable, given the methodology used for randomization and the similarity of the control and intervention groups before delivery and breastfeeding, as well as the use of appropriate statistical studies to eliminate possible confounding biases.

Finally, and to be taken into account in future lines of research, the causes of breastfeeding abandonment in breastfeeding or infants were not investigated.

## 8. Conclusions

The use of beeswax breast pads in combination with the WHO education program improved both initiation and maintenance rates of breastfeeding among first-time mothers over a 6-month follow-up period, compared to the use of the WHO health education program alone. The breastfeeding rates found at 6 months exceeded the WHO targets for 2025 and 2030. Self-perception of health status, mobility, and the sensation of pain and discomfort improved more with the use of breast areolas during breastfeeding.

## 9. Implications for Future Research

This study provides evidence to inform nursing practice and contribute to the improvement of breastfeeding mothers, thereby improving successful breastfeeding rates as well as the quality of life of breastfeeding mothers.

Possible areas of future research could be to evaluate the healing time of the cracks when using ecological beeswax breast pads versus other products on the market, as well as the implications for breastfeeding in multiparous mothers, maternal problems that lead to abandonment of breastfeeding, children with sucking problems, and in other socioeconomic environments.

## Figures and Tables

**Table 1:** Comparison of descriptive variables of the control and intervention groups before delivery.

Variable	Control Group (n = 69); n (%); Mean ± SD	Intervention Group (n = 77); n (%); Mean ± SD	*p*-Value
Age (years)	31.8 ± 4.7	31.2 ± 5.2	0.415
Goldberg Depression (pre)	1.9 ± 1.8	1.9 ± 1.8	0.930
Goldberg Anxiety (pre)	3.0 ± 2.3	3.2 ± 2.3	0.639
EQ5D mobility (pre)	1.1 ± 0.3	1.2 ± 0.4	0.118
EQ5D personal care (pre)	1.1 ± 0.3	1.1 ± 0.3	0.881
EQ5D activities of daily living (pre)	1.3 ± 0.5	1.2 ± 0.4	0.254
EQ5D pain/discomfort (pre)	1.4 ± 0.5	1.4 ± 0.5	0.508
EQ5D anxiety/depression (pre)	1.2 ± 0.4	1.2 ± 0.5	0.996
Self-perception of health status (pre)	81.9 ± 13.1	85.5 ± 13.1	0.126
Prepartum discomfort	25 (36.2)	31 (40.3)	0.617
Working outside the home	45 (66.2)	44 (57.9)	0.307
Inverted nipple	1 (1.4)	2 (2.6)	0.625
Air and sun on the chest	5 (7.2)	9 (11.7)	0.363

**Table 2:** Comparison of descriptive variables of the control and intervention postpartum.

Variable	Control Group (n = 65); n (%); Mean ± SD	Intervention Group (n = 71); n (%); mean ± SD	*p*-Value
Gestational age (weeks)	39.4 ± 1.3	39.5 ± 1.1	0.618
Vaginal birth	48 (73.8)	56 (80.0)	0.396
Breastfeeding start (hours)	1.6 ± 5.0	1.7 ± 4.8	0.966
Easy latch to the chest	48 (73.8)	55 (78.6)	0.519
Need for help	31 (47.7)	29 (37.7)	0.464
Goldberg Depression (post)	2.2 ± 2.1	1.7 ± 2.1	0.175
Goldberg Anxiety (post)	2.9 ± 2.5	2.7 ± 2.1	0.533
EQ5D mobility (post)	1.2 ± 0.4	1.1 ± 0.3	0.254
EQ5D personal care (post)	1.1 ± 0.2	1.1 ± 0.3	0.820
EQ5D activities of daily living (post)	1.3 ± 0.5	1.2 ± 0.4	0.319
EQ5D pain/discomfort (post)	1.5 ± 0.5	1.4 ± 0.5	0.307
EQ5D anxiety/depression (post)	1.3 ± 0.5	1.3 ± 0.6	0.832
Self-perception of health status (post)	77.8 ± 15.1	80.6 ± 18.6	0.354
Postpartum discomfort	43 (66.2)	50 (71.4)	0.508

**Table 3:** Analysis of primiparous mothers who continued breastfeeding at 3 and 6 months postpartum.

Variable	Control Group n/Nt (%)	Intervention Group n/Nt (%)	*p*-Value
Continued breastfeeding at 3 months	42/62 (66.7)	57/64 (87.7)	0.004
Continued breastfeeding at 6 months	36/63 (57.1)	45/59 (76.3)	0.025

**Table 4:** Binary logistic regression of first-time mothers who continued to breastfeed their infants at 3 and 6 months. * Chi-square of the model 8.363; *p* = 0.015 ** chi-square of the model 4.773; *p* = 0.092.

	Breastfeeding at 3 Months *		Breastfeeding at 6 Months **	
Variable	Adjusted OR 95% CI	*p*-Value	Adjusted OR 95% CI	*p*-Value
Type of delivery (vaginal vs. cesarean)	0.561 (0.211–1.490)	0.246	0.814 (0.324–2.045)	0.662
Use of breast pads	3.129 (1.249–7.839)	0.015	2.282 (1.038–5.016)	0.040

## Data Availability

The raw data supporting the conclusions of this article will be made available by the authors on request.
